# Three-dimensional motion analysis for comprehensive understanding of gait characteristics after sciatic nerve lesion in rodents

**DOI:** 10.1038/s41598-018-31579-z

**Published:** 2018-09-11

**Authors:** Junichi Tajino, Akira Ito, Momoko Tanima, Shoki Yamaguchi, Hirotaka Iijima, Akihiro Nakahata, Wataru Kiyan, Tomoki Aoyama, Hiroshi Kuroki

**Affiliations:** 1 Department of Motor Function Development and Rehabilitation, Human Health Sciences, Graduate School of Medicine, Kyoto University, 53 Shogoin Kawahara-cho, Sakyo-ku, 606-8507 Kyoto Japan; 2 Department of Motor Function Analysis, Human Health Sciences, Graduate School of Medicine, Kyoto University, 53 Shogoin Kawahara-cho, Sakyo-ku, 606-8507 Kyoto Japan; 3 Medical Genetics Branch, National Human Genome Research Institute, National Institutes of Health, Bethesda, MD USA; 4 Department of Physical Therapy, International University of Health and Welfare, Chiba, Japan; 5 Department of System Design Engineering, Keio University, Tokyo, Japan

## Abstract

Rodent models of sciatic nerve lesion are regularly used to assess functional deficits in nerves. Impaired locomotor functions induced by sciatic nerve lesion are currently evaluated with scoring systems despite their limitations. To overcome these shortcomings, which includes low sensitivity, little significance, and the representation of only marginal components of motion profiles, some additional metrics have been introduced. However, a quantitative determination of motion deficits is yet to be established. We used a three-dimensional motion analysis to investigate gait deficits after sciatic nerve lesion in rats. This enabled us to depict the distorted gait motion using both traditional parameters and novel readouts that are specific for the three-dimensional analysis. Our results suggest that three-dimensional motion analysis facilitates a comprehensive understanding of the gait impairment specifically, but not limited to, a sciatic lesion rat model. A broad application of these methods will improve understanding and standardized motor assessment.

## Introduction

Animal models are widely used to study nerve damage. To address the functional deficits as consequences of these nerve lesion, motion analysis of animals is common and supposed to represent the characteristics of neural malfunctions^1–3^. Among the several parameters for assessment, including learning abilities^4^, somatosensory^5^, and endurance^6^, kinematic gait analysis is considered a promising method for addressing the motor aspects of impairment; it is even more sensitive than the conventional metrics, despite being labor-intensive^3,7^. Several apparatus and methodologies are currently available for gait analysis (e.g., video recording in the sagittal plane^8^ and radiographic imaging^9^). Zorner *et al*. explored two-dimensional (2D) metrics that could represent the motion deficits in spinal cord injury (SCI), stroke, and transgenic (Epha4^−/−^, results in “hopping” hind limbs) mice^10^. Ueno *et al*. utilized a three-dimensional (3D) reconstruction model to address the asymmetry in the gait of mice with lesions in their motor cortex^11^. In regard to the peripheral nerve damage models, sciatic nerve lesions are often studied to investigate its pathological symptoms and the efficacy of relevant remedies. Among the impairments induced by sciatic nerve lesions including antalgic, sensory, and paretic aspects, animal gait kinematic assessment principally has advantages to represent motor paralysis^12–14^. It could also overcome the short comings and reinforce the significances of the sciatic functional index (SFI), which is still the gold standard for the sciatic nerve lesion model^15^. However, most of those video-based readouts are specific to certain motor deficits and investigators often have to re-establish a new assessment protocol that is specific to each type of malfunction, as previous studies have addressed^10,16^. The lack of a systematic, standardized, and reliable method would be an obstacle for a wider application of animal gait kinematic analysis. In this study, we explored the advantages of gait kinematic analysis of rats in order to address the standardized assessment of motion in rodents. We also examined 3D reconstructions in order to make full use of the 3D assessment apparatus and provided suggestions for novel parameters.

## Results

### Overview

In this study, we attempted to elucidate the possibility of the 3D analysis of rodent gait model. This time, we addressed a rat sciatic nerve defect model with intention to further characterize the motor paralysis of this model. We explored a set of metrics using 3D gait characteristics reconstructed from a multi-camera recording of their walking. Gait motions were detected by using small plastic markers for tracking that were attached at anatomical landmarks. To illustrate the unique features of 2D and 3D analysis, we further categorized those parameters into subgroups.

The 2D parameters that we measured included angle excursions of the ankle and toe within one-step cycle. Based on these values, we obtained joint trajectories and ranges of motion (ROMs) as the basic features of spatial parameters. Subsequently, we evaluated ankle stance angle (ASA, ankle angle at midstance) as a spatial parameter, as previously described^17^. We also suggested these additional spatial parameters as follows: ankle to the ground (AtG), ankle angle to the ground at midstance; metatarso-ankle distance (MAD), distance between the ankle and metatarsophalangeal joint (MTP) markers at midstance; AoA (angle of attack), toe angle at the final segment of the swing phase^18^. To further characterize the relationship between these two joints, we also drew Lissajous graphs of the toe and ankle accompanied with a circular plot. For the temporal parameters, we evaluated joint angle acceleration and range of angular acceleration (RAA) for each joint in order to explain the dynamic properties.

For the 3D specific parameters, we obtained the toe out angle (TOA, the angle of the toe against the progression line of the animal in the horizontal plane) by calculating the 3D reconstructed properties, which was originally based on foot prints from previous studies^19^. We also calculated the knee out angle (KOA, the angle of thigh in the same dimension as the TOA), which is only available through a 3D analysis. In addition, in order to standardize the measurements, we re-measured the TOA and KOA using different methods (toe and hip abduction, respectively; relative to the body axis of the animal instead of the progression line).

As unique parameters of 3D analysis, we further evaluated the pelvic properties, which consists of a pelvic shift (right-left displacement), pelvic tilt (rolling angle), pelvic rotation (yawing angle), and pelvic travel, which is travel area of the center of gravity (CoG; the area surrounded by the trajectory of the CoG of the pelvis in the coronal plane). We have chosen these 3D parameters this time since paretic aspect of sciatic nerve lesion is well represented by impaired ankle/toe motion and consequential instabilities of proximal portions^16^. However, it does not necessarily mean that general application of other kinematic parameters is limited.

### Findings in the gait motion analysis

**Figure 1:**
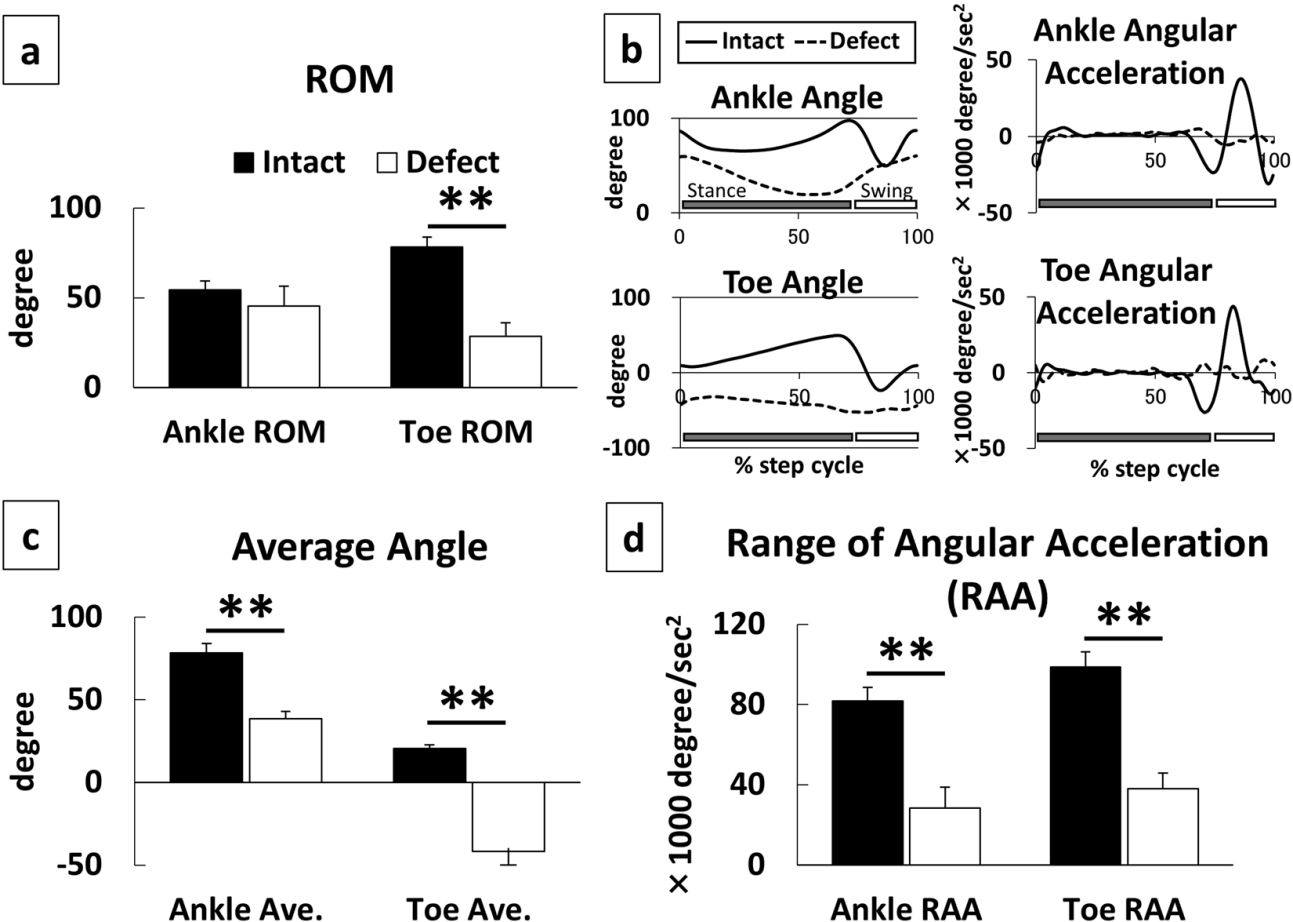
Basic features of joint motion derived from joint trajectories (excursions) during one step cycle. Range of Motion (ROM) (**a**), Average Angle (**c**), Range of Angular Acceleration (RAA) (**d**), Time Course Trajectories of Joint Angle and Acceleration (**b**). One step cycle spans between a paw contact to the next contact of the same paw (n = 6 for each group). Nerve Defect group showed significant decrease in each metric (**a,c,d**) except for Ankle ROM (**a**), despite the Ankle joint trajectory showed distinct decline in Defect group (**b**). **p < 0.01 to Intact (unpaired t test).

Nerve defect group showed a significant decline in performance of almost all the evaluated parameters compared to those of the intact group. Joint trajectories of ankle and toe visualize decreased motions (Fig. 1b). More flexed angle during stance phase (Fig. 1b left panels) implies impaired weight bearing of ankle, inappropriate contact of paw, and absence of push off. Flat state of acceleration during swing phase (Fig. 1b right panels) implies lack of foot clearance motions. These deficiencies were further quantified with a series of following parameters.

#### 2D parameters

**Figure 2:**
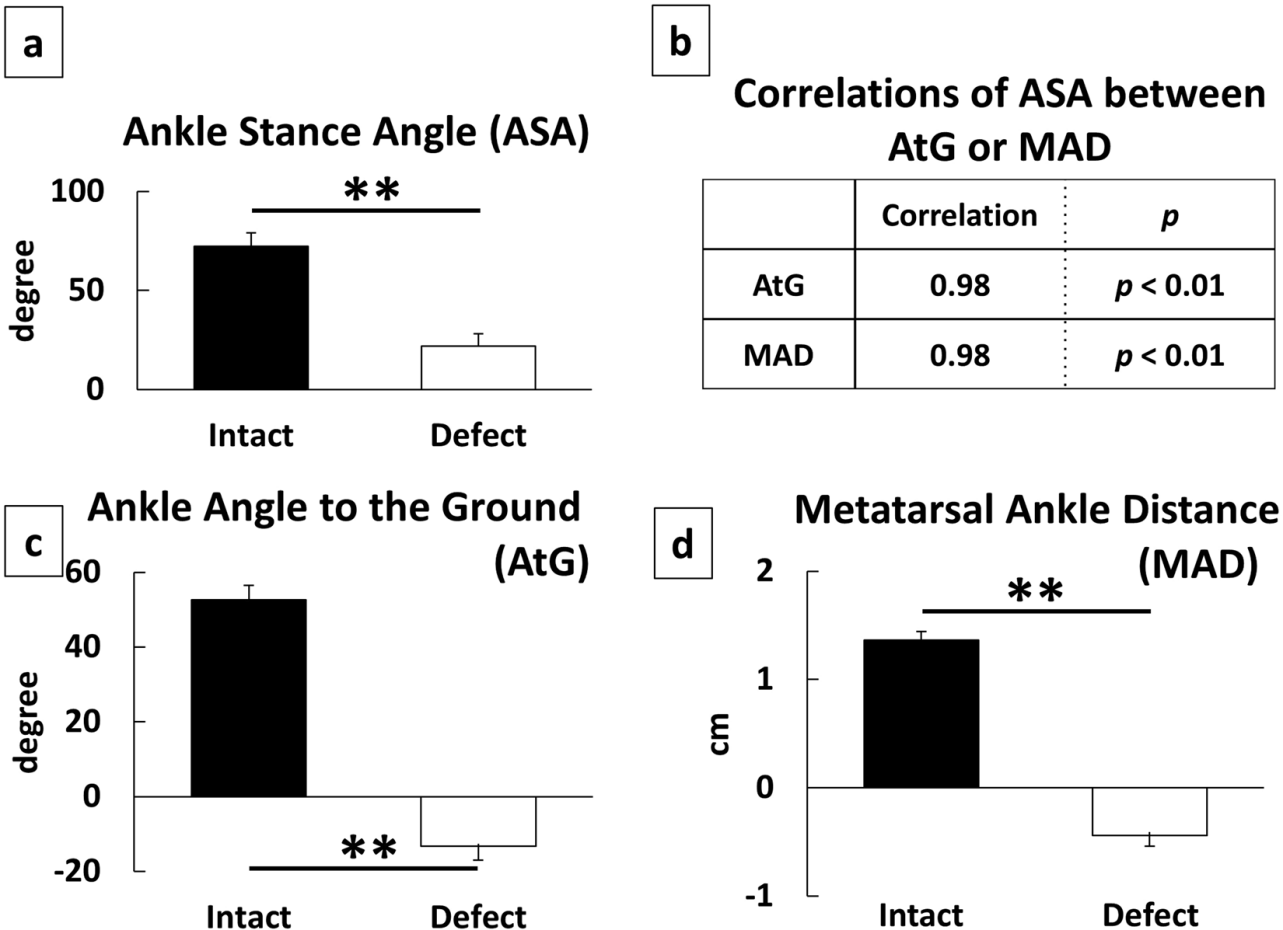
Characteristics of ankle joint at mid stance. Ankle Stance Angle (ASA) as described in previous studies (**a**), Ankle Angle to the Ground (AtG), and Metatarsal Ankle Distance (MAD) which are based on ground surface (**c** and **d**, respectively), and correlations of AtG and MAD to ASA (**b**) (n = 6 for each group). Nerve Defect group showed significant reductions in each parameter (**a,c,d**). In addition, both AtG and MAD had high correlation between ASA (**b**). **p < 0.01 to Intact (unpaired t test).

**Figure 3:**
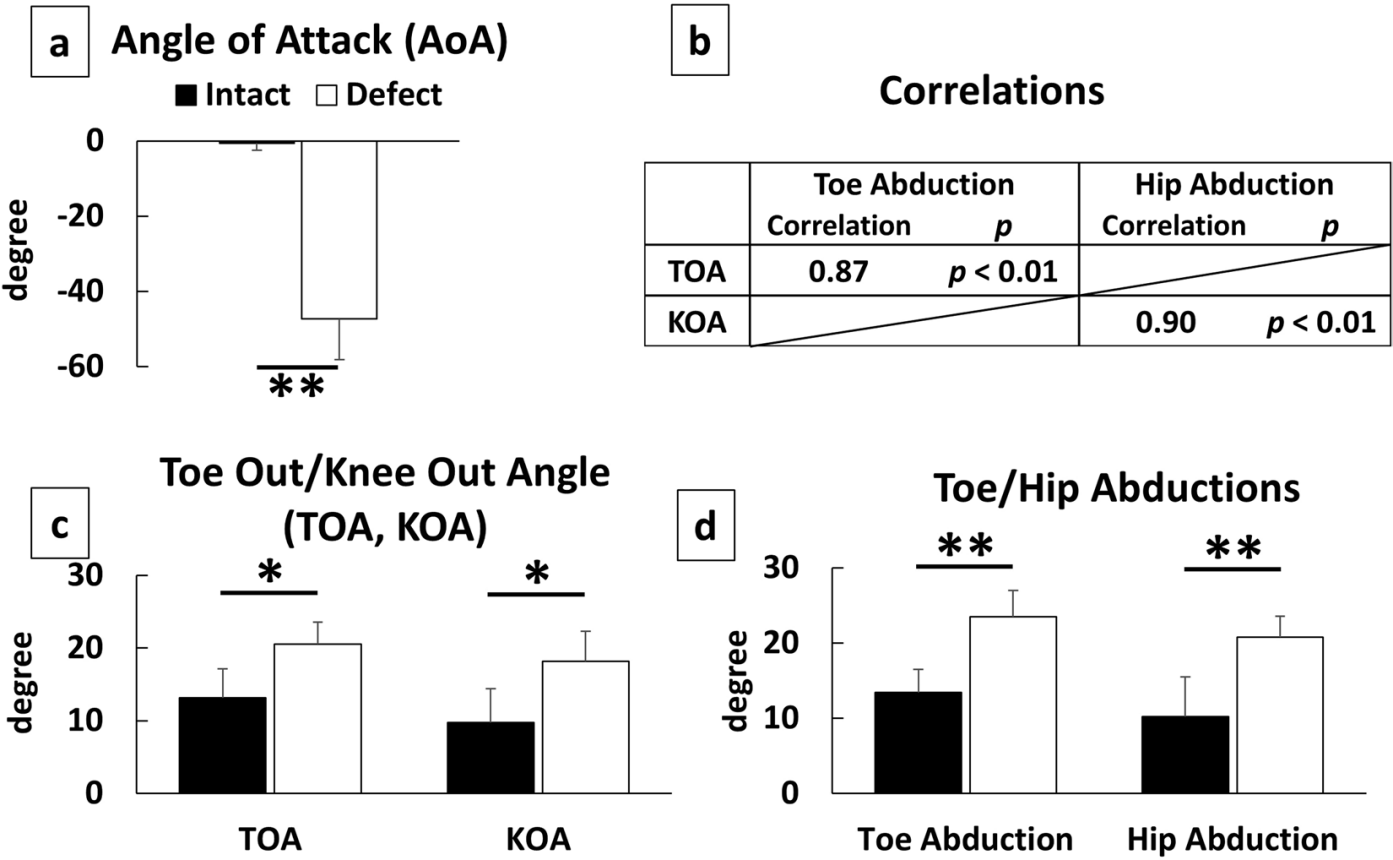
Characteristics of toe and knee. Angle of Attack (AoA) which is toe angle at the final segment of the swing phase (**a**), Toe Out and Knee Out Angles (TOA, KOA) based on the progression line of the animal (**c**) and equivalents of those based on body axis of the animal (Toe/Hip Abductions) (**d**) (n = 6 for each group). Correlations of TOA between Toe Abduction and KOA between Hip Abduction (**b**) (n = 6 for each group). There were significant differences between groups in AoA (**a**), TOA/KOA (**b**), and Toe/Hip Abductions (**c**) respectively. KOA, Toe/Hip Abductions are only available via three-dimensional analysis since the reference line of femur (for KOA) and body axis for the animals (for Toe/Hip Abductions) have to be reconstructed from captured data. High correlations between equivalent parameters (TOA with Toe Abduction; KOA with Hip Abduction) suggest that the parameters based on the body axis also effectively represent the extra abductions of their feet and legs. Metrics based on the body axis might be more suitable to express the joint motion since it is not affected by the direction of locomotion. *p < 0.05 to Intact (unpaired t test). **p < 0.01 to Intact (unpaired t test).

**Figure 4:**
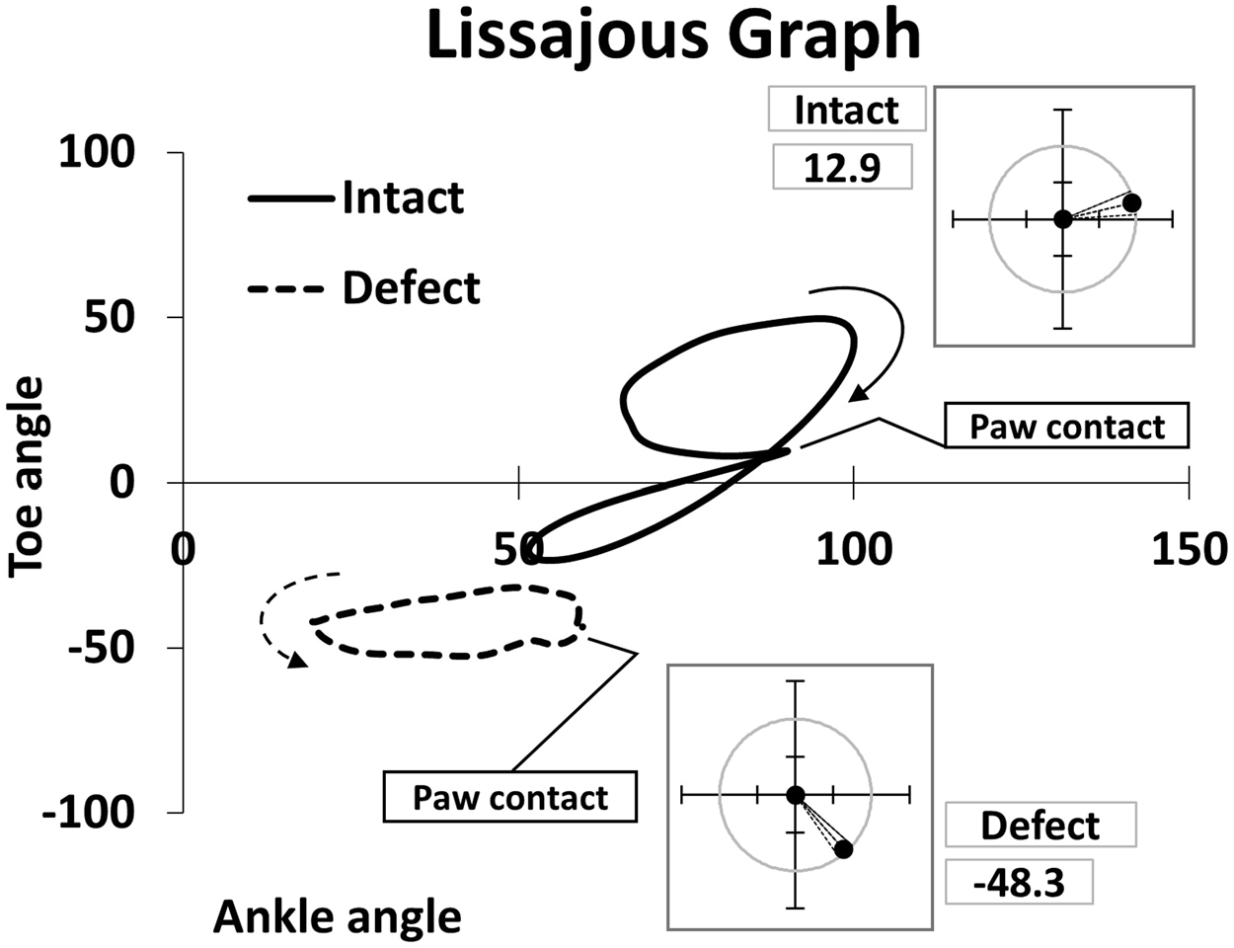
Lissajous Graph of toe and ankle joint. Inset circular plots show in what quadrant each Lissajous lies in a form of angle that sets positive x axis as the reference line (n = 6 for each group). Defect group showed more flexed and limited motion ranges especially in toe. Despite the difficulties for statistical determination, Lissajous graph would be useful to visualize the motion profile.

For the ROM and the average of joint angles during the one-step cycle, results of a unpaired *t* test showed that the nerve defect group had a significant decrease (*p* < 0.01) in all parameters than did the intact group, except the ROM of the ankle (*p* > 0.05; Fig. 1a,c). For the other spatial parameters at midstance, ASA, AtG, and MAD, exhibited significant decrease in the nerve defect group than in the intact group (*p* < 0.01; unpaired *t* test; Fig. 2a,c,d). In addition, AoA, which represents the functional state of the toe during the swing phase, was significantly lower in the nerve defect group when compared to that of the intact group (*p* < 0.01; unpaired *t* test; Fig. 3a). Results from the Lissajous and circular plots showed a significant difference between the two groups (*p* < 0.01 for the circular plot (insets); unpaired *t* test; Fig. 4).

For the temporal parameters (RAA), the nerve defect group showed a significant reduction in both ankle and toe than those of the intact group (*p* < 0.01 for both, unpaired *t* test; Fig. 1d).

#### 3D specific parameters

Both TOA and KOA of the nerve defect group were significantly larger than those of the intact group (*p* < 0.05 for both, unpaired *t* test; Fig. 3c). In addition, toe abduction and hip abduction were significantly increased in the nerve defect group (*p* < 0.01 for both, unpaired *t* test; Fig. 3d).

**Figure 5:**
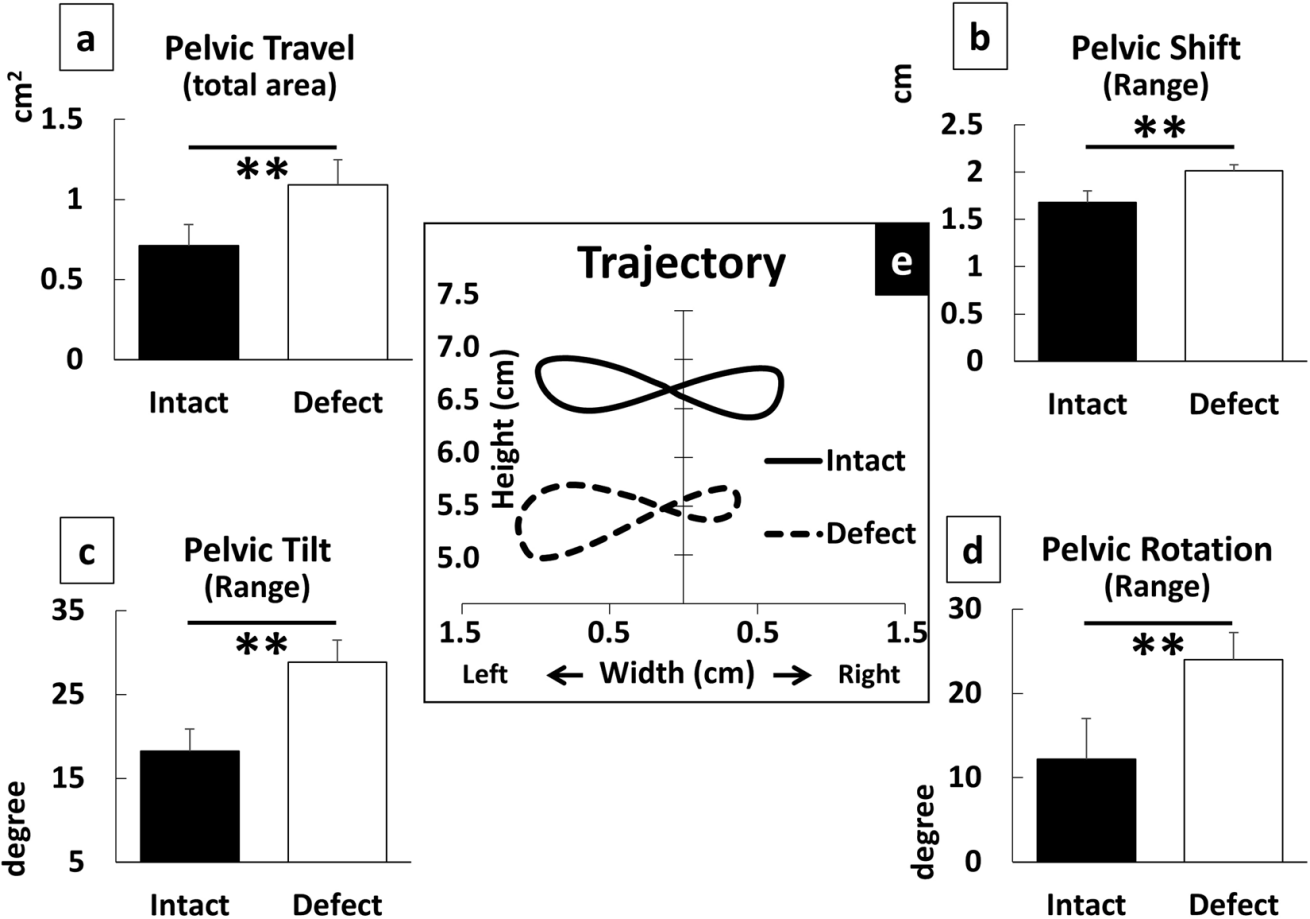
Pelvic Properties based on reconstructed features of pelvic motion. They represent the range of horizontal shift in the coronal plane (**b**), roll tilt (**c**), and yawing rotation (**d**) (n = 6 for each group). They showed significant difference between groups. Further, the reconstruction data enabled us to characterize the motion of COG (Center of Gravity) of the pelvis that showed asymmetry (**e**) and significant increase in the projected area (**a**) in the coronal plane. **p < 0.01 to Intact (unpaired t test).

For the pelvic properties, all results from the analysis of the subparameters in the nerve defect group were significantly higher than those of the intact group, suggesting that when a nerve defect is present, the motion of the pelvis was less stable (*p* < 0.01 for each, unpaired t test; Fig. 5a–e).

#### Comparison between conventional parameters

We determined the correlations between the conventional (ASA, TOA, KOA) and our novel parameters (MAD and AtG for ASA; toe abduction for TOA; hip abduction for KOA, respectively) to validate the new parameters. Our results showed a strong correlation between the conventional and new parameters (Pearson’s correlation coefficients = 0.87–0.98, *p* < 0.01; Fig. 2b, AtG and MAD for ASA; Fig. 3b, toe abduction and hip abduction for TOA and KOA).

## Discussion

We intended to propose comprehensive and quantitative method for the analysis of 3D gait motion in rat peripheral nerve defect models through the introduction of novel parameters that correlated with the conventional parameters. Our results revealed a significant difference in the walking motions between the intact and sciatic nerve defect rat models. In addition, among the novel parameters we suggested, some are unique to 3D analysis and further characterize the specific properties of motion in the nerve defect group. They are also consistent with the conventional parameters.

ROM of ankle was not significantly different between the two groups. This implies that simply measuring the spatial aspects of the joints does not always represent the motor functions of the joints. An explanation is that the ankle joint might be moved by momentum, which could also represent the toe dragging in the nerve defect group, as shown in a previous study^18^.

Due to the inertial pseudomotion of the foot segment and the fact that the ankle joint does not touch the ground during the stance phase of intact walking, it would be more suitable to measure the relationship between the ankle and ground surface rather than angle of ankle joint itself. Both AtG and MAD showed a significant difference between the groups and were correlated with the ASA. This might suggest that AtG and MAD are more precise representations of the state of ankle joint. The strong correlation of AtG and MAD with ASA (Fig. 2b) supports this interpretation.

To ensure a safe landing of the paw, the toes must be extended and spread before paw contact^16^. We call this event AoA and it represents the function of toe in conjunction with its acceleration^20^. Even when the weight bearing motion (during stance phase) has not yet restored, the motion during the swing phase, which is non-weight bearing, could appear earlier than those of the stance phase during the recovery. This paretic state is one of particular profiles of sciatic nerve lesion model and significance of the assessment of toe-spread during the swing phase is also addressed by Fey *et al*.^16^. Assessment of recovery in AoA could make the evaluation of nerve lesions more reliable.

**Figure 6:**
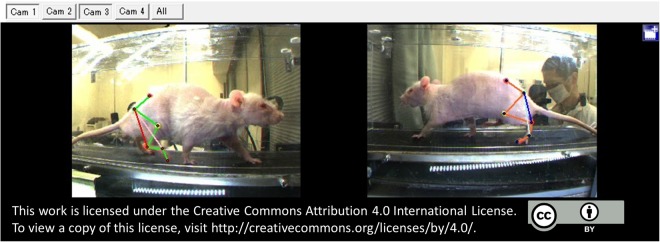
Overall diagram of data acquisition. Kinematic data was obtained while the animal was walking on the treadmill at 20 cm/sec.

We have incorporated Lissajous plot to further represent the joint motions. The Lissajous plot of ankle and toe motions represents not only the extent of motion of an individual joint, but also the mutual relationship between them. A narrow Lissajous plot that lies in the sub-zero quadrant along the y-axis (as the inset circular plot in Fig. 6 shows) suggests less motion with a more flexed toe throughout the step cycle. To the best of our knowledge, this is the first study that uses a Lissajous plot of the ankle and toe to analyze movement in the context of a nerve lesion. This metric would provide visual insights of joint motions and could be applied to other joints, although the statistical interpretation is still difficult.

Joint angle acceleration and RAA could represent the temporal aspect of motion, in addition to the spatial aspect represented by the ROMs. In this study, ankle ROM of the nerve defect group was similar to those of intact group, despite the obvious impaired voluntary motion seen in the nerve defect group. This inconsistency is also supported by discriminant analysis among the parameters (Table 1). Measuring the joint angle acceleration has an advantage to describe this voluntary aspect of motion. This might imply that observing both ROM and RAA would provide a more precise representation.

**Table 1:** Discriminant Analysis to explore the ability to destinguish two groups.

		F	*p*			F	*p*			F	*p*
ROM	Ankle	2.1	>0.05	Average angle	Ankle	116.6	<0.01	RAA	Ankle	70.8	<0.01
	Toe	112.9	<0.01		Toe	133.1	<0.01		Toe	120.4	<0.01
ASA		117.0	<0.01	AtG		583.5	<0.01	MAD		759.8	<0.01
AoA		68.4	<0.01	TOA/KOA	TOA	8.3	<0.05	Abductions	Toe A.	18.4	<0.01
					KOA	7.0	<0.05		Hip A.	12.0	<0.01
		**F**	***p***		**F**	***p***					
Pelvic Properties	Travel	12.0	<0.05	Tilt	12.4	<0.05					
	Shift	11.1	<0.05	Rotation	16.7	<0.01					

We attempted to introduce novel parameters using 3D motion analysis in a nerve defect model in rodents. Unlike the results of a foot print analysis or a 2D motion analysis, results from a 3D analysis allow us to do the following: (1) set the reference or observation line using imaginary lines not only lines that are in contact with the ground and (2) observe the time course change throughout the step cycle. It enabled us to analyze the KOA, which is addressed using the line of the femur reconstructed out of 3D data. In addition, we could address toe abduction and hip abduction, which are the equivalent to TOA and KOA, but use the body axis of the animal instead of the progression line as the reference. Since animals do not always walk straight forward in the experimental corridor, it might be better to refer to the body axis rather than the progression line. The strong correlations of TOA with toe abduction and KOA with hip abduction (0.87 and 0.90, respectively) show that these new parameters also effectively represent the extra abductions of their feet and legs. Referring to the imaginary body axis, not only the progression line, might be more suitable to express the leg motion since it is not affected by the direction of locomotion. In addition, 3D analysis can track the change in the time course of the parameters during a step cycle. Although it was not a main focus of this study, it seemed that TOA (toe abduction) of intact gait increased through the stance phase after the paw contact (data not shown).

We also implemented pelvic properties because they are uniquely possible by 3D analysis. Pelvic properties consists of the following four subsets: pelvic shift; tilt; rotation; and pelvic travel. They could represent the coronal asymmetry of walking, not only of limb joint excursions. It is interesting that the nerve defect group showed more elevation than the intact group on the affected side; however, this is reasonable given the compensatory movement for the potential toe drag during the swing phase. Gait asymmetry is one of characteristics of sciatic nerve lesion model. Pelvic properties would be useful to describe this profile as well as distinguish this nerve lesion model from other motion disorders.

Despite these advantages of 3D motion analysis, this study still has limitations. The results might be affected by the fact that different animals were used for each group. However, considering that the ages of the animals were almost identical, we believe the difference in the characteristics of motion had a minor impact. Although we compared some of novel parameters with the conventional ones, we did not compare them to conventional foot print analysis including SFI. This will need to be validated in future studies.

The findings in this study suggest a potential advantage with 3D motion analysis and also highlight the need for detailed locomotor profiling in rodent studies. For the sciatic nerve lesion model that we used this time, these kinematic analyses have advantages to represent the paretic aspect of impairments. Among them, our parameters showed potential benefits to further characterize the state of recovery, voluntary motion as well as distinct motion of this model. The application of systematic and quantitative metrics might improve and help standardize the assessment of animal motions.

## Methods

### Animals

All experiments were performed with approval of and in accordance with guidelines of the Kyoto University Animal Experimentation Committee. We used two groups of rats (a total of twelve animals; 210–250 g). Six adult male F344-rnu/rnu rats with an immune deficiency were used for the Nerve Defect group. For the control (Intact group), six male Wistar rats were used. The animals were 8–9 weeks old at the beginning of the study. All animals were housed in groups of two per cage with a 12 h:12 h light:dark cycle and took food and water ad libitum.

### Surgery and animal care

For the nerve defect group (n = 6), we performed nerve defect surgery in the sciatic nerve. Under general anesthesia using an intraperitoneal injection of 0.85 ml/kg somnopentyl, the sciatic nerve of each rat was exposed through a longitudinal skin incision in the gluteal region of their right thigh. The sciatic nerve was cut to create a 5-mm interstump gap between the proximal and distal end of the nerve. We administered 0.02 mg/kg fentanyl intraperitoneally for postoperative analgesia. Subsequently, all animals in both groups were allowed to walk freely in their cages until data acquisition.

### Three-dimensional motion capture apparatus

To obtain and process the kinematic properties of locomotion, we used a 3D motion capture apparatus (Kinema Tracer System; Kissei Comtec, Nagano, Japan). This system is composed of 4 charge coupled device cameras (CCD cameras) and the software components used to process the data. In addition, the setup for this study contained a treadmill, which was enclosed inside a Plexiglas basin (height, 220 mm; width, 115 mm; length, 630 mm), for the rodents. Four cameras were placed so that two of them were in-line on the one long side of the basin and the other two cameras were on the other side. The apparatus captured a 120-Hz image of hind limb motion during walking, which enabled a 3D reconstruction of the movements^21^.

### Preparation of animals and data acquisition

Before each motion capture session, rats were bilaterally equipped with joint markers on their hindlimbs^22^. We used plastic markers or paint ink to mark the following 6 landmarks on each hind limb: anterior superior iliac spine (ASIS); trochanter major (hip); knee joint (knee); lateral malleolus (ankle); fifth metatarsophalangeal joint (MTP); and distal tip of middle toe (toe). For the five proximal landmarks, we attached colored hemispherical plastic markers (3 mm in diameter) onto the shaved skin overlying the defined landmarks. Briefly, hair around hindlimbs was shaved and bony landmarks were palpated while the animal was under light inhalational anaesthesia. The experimenter manually made slight flexions and extensions of the joints to confirm the coordinates. Subsequently, landmarks were targeted with black ink and plastic markers were attached. As for the toe, we used acrylic ink for marking (paint marker, NIPPON PAINT Co., Ltd, Tokyo Japan). Hind limb motion was captured while the rats were walking at 20 cm/sec on the treadmill (Fig. 6). A total of 10 steps from trials during which the animal walked at least 5 consecutive steps were incorporated to the data sets of each rat^9^. Subsequently, markers were automatically tracked and the 3D properties were reconstructed by the system based on the coordinates. We calculated the set of parameters for the current study using pre-formatted Excel spreadsheets (Microsoft Office) or MATLAB scripts (MathWorks, Boston MA USA). To ensure data accuracy, we calibrated the precise coordinates of a standard object (height, 100 mm; width, 50 mm; length, 200 mm) prior to each session.

### Motion parameters in locomotion

We evaluated several parameters to depict the gait motion of the nerve defect model. Subsequently, we categorized them into two groups: sagittal (2D) and 3D specific parameters. The sagittal (2D) group includes metrics that can be observed in the sagittal plane including spatial and temporal parameters. The 3D specific group refers to those in the coronal or horizontal planes that are specific to the 3D reconstructed components.

### 2D parameters

2D parameters were derived from the ankle angle and toe angle during gait cycles. We defined ankle angle by two segments, one referring to the shank and another connecting the ankle and fifth MTP. For the toe angle, we defined as the angle between fifth metatarsal bone and the segment connecting fifth MTP and the tip of the middle toe. For these (ankle/toe) angles, greater angles refer to extension (plantar flexion) for ankle and extension (dorsiflexion) for phalanges, respectively (please see Table 2 for details). Joint trajectories and ROMs were acquired as basic features of spatial parameters. We derived the other spatial parameters using the following methods: ASA was determined as previously reported^17^; AtG was the angle between the ground surface and fifth metatarsal bone at midstance; MAD was the vertical height of ankle minus those of MTP at midstance; AoA was the angle between the toe and metatarsal bone at the final segment of swing phase^18^.

**Table 2:** Description of joint movements.

		Increase	decrease	negative value implies
Joint	Ankle	plantar flex	dorsiflex	
Angles	Toe	extension of phalanges	flexion of phalanges	distal ends of phalanges are below the metatarsal bones
RAA	Ankle	wider transition in velocity	less transition in velocity	
	Toe			
ASA		plantar flex of ankle	dorsiflex of ankle	
AtG		metatarsal bone upright	metatarsal bone laid down	distal end of metatarsal bone is not on the ground
MAD		ankle joint is elevated	ankle joint is dropped	ankle is below the distal end of the metatarsal bone
AoA		extension of toe phalanges	flexion of toe phalanges	Further negative value implies less functional spread of the toe before the paw contact.

Midstance was defined as the moment when the relevant leg was passed by the opposite swinging leg^17^ or hip marker of the relevant leg was in-line with the MTP in the vertical plane. The final segment of swing phase was defined as the moment when the MTP was in the vertical plane beneath the knee joint during swing phase. For a better understanding of the relationship between the angles of the ankle and toe, we also generated Lissajous graphs of these two joints with an inset circular plot that refers to the relevant quadrant.

For temporal parameters, we suggested a joint angle acceleration and RAA. These temporal parameters enabled us to comprehend the dynamic properties of each joint.

### 3D specific parameters

3D parameters were derived from the reconstructed properties of processed data, which enabled us to further illustrate the characteristics of the motion. We evaluated them as follows: TOA was the angle between the direction of progression and the reference line of the foot^19^, KOA was the angle between the direction of progression and the reference line of the thigh, toe abduction was the angle between the body axis and the reference line of the foot (defined as external rotation of the hind limb in a study by Lin FM *et al*.^17^), and hip abduction was the angle between the body axis and the reference line of the thigh. The reference lines representing the foot and thigh were defined as the line connecting the ankle and toe markers and the line connecting hip and knee markers, respectively. The body axis was referred to the line in the horizontal plane that was perpendicular to the virtual line connecting the right and left anterior superior iliac spine (ASIS).

Furthermore, we implemented a series of evaluations of animals’ body movements that was directly/virtually acquired out of the coordinates. We collectively call it pelvic properties. Pelvic properties consisted of a series of the following subparameters: pelvic shift, the deviation of a virtual marker which located in the midpoint between right and left hip markers in the coronal plane; pelvic tilt, the angle between the horizontal line and the line connecting right and left hip markers (also defined as midline deviation by Yu *et al*.^8^); pelvic rotation, the angle between the progression line and the body axis in the horizontal plane; travel area of the CoG, the area surrounded by the trajectory of the CoG of the pelvis in the coronal plane. CoG was virtually obtained from right and left ASISs and hip joints for each recording frame (120 Hz).

### Statistics

We used unpaired *t*-tests to determine if differences in the parameters between intact and nerve defect groups existed. We used Pearson’s correlation coefficients (between ASA vs. AtG, MAD; TOA vs. Toe Abduction; KOA vs. Hip abduction) to determine the strength of the correlation. All tests were performed with JMP Pro Version 12 (SAS Institute Inc., Cary NC, USA). Data and graphs are represented as group means and error bars represent the 95% confidence interval. Statistical significance was set to *p* < 0.05.

## Data Availability

The data generated during and/or analysed during the current study are available from the corresponding author on reasonable request.
